# HETEROTOPIC OSSIFICATION AFTER SPINAL CORD INJURY: PREVENTION AND TREATMENT - A SISTEMATIC REVIEW

**DOI:** 10.1590/1413-785220233103e267451

**Published:** 2023-07-17

**Authors:** CINDY YUKIE NAKANO SCHINCARIOL, EDUARDO MARTIN INSFRÁN ECHAURI, ORCIZO FRANCISCO SILVESTRE, ALBERTO CLIQUET

**Affiliations:** 1 Universidade Estadual de Campinas, Departamento de Ortopedia, Reumatologia e Traumatologia, Campinas, SP, Brazil.

**Keywords:** Spinal Cord Injuries, Ossification, Heterotopic, Therapeutics, Traumatismos da Medula Espinal, Ossificação Heterotópica, Terapêutica

## Abstract

Trauma configures the main cause of spinal cord injuries. Patients with traumatic spinal cord injury often develop severe and debilitating outcomes that require multidisciplinary care to adapt patients to their new reality. Heterotopic ossification (HO) is one of the frequent comorbidities in these patients but it still lacks well-established treatments or a gold standard one. Thus, this systematic review aimed to search the current literature for HO treatment and prevention. This study was conducted following PRISMA recommendations (Preferred Reporting Items for Systematic Reviews and Meta-Analysis) and searches were conducted in three databases (PubMed, Embase, and Web of Science). A total of 193 articles were found in an initial search. After screening following the established criteria, eight articles were included in this review; of these, two reported prevention and the others, treatments. Based on data analysis, the use of non-steroidal anti-inflammatory drugs in the acute post-traumatic period proved to be the best method of prevention. In cases of mature HO or accompanied by ankylosis, surgical resection proved to be the most effective treatment despite the high rate of postoperative infections. ***Level of Evidence III, Systematic Review.***

## RESUMO

A principal causa de lesão medular é o trauma. O paciente com trauma raquimedular frequentemente evolui com incapacidades graves e debilitantes, fazendo com que necessite de cuidado multiprofissional para se adaptar a uma nova realidade. A ossificação heterotópica (OH) é uma comorbidade comum nesses pacientes, mas que ainda não tem tratamentos bem estabelecidos ou considerados padrão-ouro. Esta revisão sistemática teve como objetivo buscar formas de tratamento e prevenção da OH na literatura atual. O estudo seguiu a recomendação Principais Itens para Relatar Revisões Sistemáticas e Meta-Análises (PRISMA) e realizou buscas em três bases de literatura (PubMed, Embase e Web of Science). Foram encontrados 193 artigos inicialmente, e, após triagem considerando os critérios estabelecidos, oito foram eleitos para o trabalho final. Dois trabalhos abordaram a prevenção; e os demais, tratamentos. Pela análise dos dados, o uso de anti-inflamatórios não esteroidais (AINEs) no período agudo pós-traumático se mostrou como o melhor método de prevenção. Já em relação ao tratamento, nos casos de OH maduras ou com anquilose, a ressecção cirúrgica revelou-se como o método mais efetivo, a despeito da grande taxa de infecção pós-operatória. **Nível de Evidência III, Revisão Sistemática.**

## INTRODUCTION

Spinal cord injuries (SCI) constitute an important economic and public health problem, mostly affecting the younger population. Its annual worldwide incidence revolves around 15 to 40 cases per million population. Motor vehicle accidents, violence, recreational activity, and falling from heights feature among the causes of this injury.^1^

The United States showed an incidence of traumatic spinal cord injury in 2017 of about 54 per million people per year and a prevalence of 280,000 survivors. ^(^ ^2^

Population aging increases the mean age of patients at the time of trauma but plays no proportional role in their survival due to the high morbidity and mortality of this patient group, especially after 60 years. ^(^ ^3^

Such an event becomes more devastating due to the chronic and irreversible changes it brings to survivors, ^(^ ^4^ including musculoskeletal changes, especially heterotopic ossification and disuse osteoporosis.

Heterotopic ossification (HO) configures an anomalous bone deposition in periarticular soft tissues. It occurs mainly in the hips^5^ and affects more than half of those with spinal cord injuries, with an average onset of three months after the injury. ^(^ ^6^

The most common clinical picture include reduced range of motion of the joint with periarticular edema. It may also be associated with pain, erythema, localized increased temperature (when sensitivity is preserved), and low-grade fever. ^(^ ^7^

The literature still lacks a consensus of a gold standard to treat neurogenic HO, mentioning non-steroidal anti-inflammatory drugs (NSAIDs), bisphosphonates, radiotherapy (RT), and resection surgery. ^(^ ^8^ ^),(^ ^9^

Therefore, we find a growing demand for the best treatment and prevention of these chronic changes in people with spinal cord injuries, aiming at a better quality of life and possible increased survival. This review aimed to search the current literature for proposals to treat and prevent HO.

## METHODS

### Search strategy

This review was conducted following PRISMA (Main items to report Systematic Reviews and Meta-analyses) guidelines. ^(^ ^10^ To design our search strategy, the PICO framework (Patient, Intervention, Control, Outcome) was initially used to construct a suitable guiding question. “What is the optimal treatment or prevention for heterotopic ossification in patients with spinal cord injuries?” Then, the following descriptors and Boolean operators were selected for our search strategy: “Spinal cord injuries” AND; “Ossification, heterotopic” AND (Therapeutics OR Prevention), with the selection filter set for articles published in the last 10 years. Searches were conducted in three databases: PubMed, Embase, and Web of Science.

### Selection criteria

Our inclusion criterion consisted of 1) original articles dealing with treatment or prevention of neurogenic heterotopic ossification of traumatic origin, whereas our exclusion criteria, of 1) non-neurogenic heterotopic ossification; 2) review articles; 3) articles that aren't mainly focused on treating or preventing HO; and 4) case reports.

### Analyzed data

The following data were extracted: author and year of publication, study design, sample size, intervention, incidence of HO solution/recurrence rate, and intervention-related complications.

## RESULTS

### Search results

**Figure 1:**
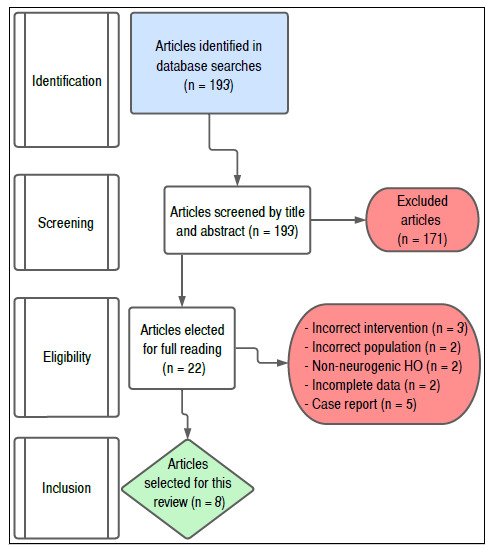
Flowchart. HO: heterotopic ossification.

We found 125 Studies on PubMed, 68 in Embase, and zero in Web of Science, totaling 193 articles (Figure 1) from the research we conducted on 05/31/2022. We found no duplicates.

We excluded studies by title and abstract in the first screening phase. The first and second reviewers independently excluded 170 studies, totaling 23 selected articles, 11 of which showed conflicts. The conflict was resolved by a third reviewer. We chose 22 for our second review stage, in which we were to fully read them. Then, we chose eight articles for this study, excluding 14 according to our selection criterion.

### Characteristics of the included studies

All articles were retrospective studies, five of which were cohort studies and three, case controls. From these, two studies dealt with HO prophylaxis (one with bisphosphonate and the other with NSAIDs) and the others, with treatment (surgical resection, radiotherapy, and combinations of techniques). Table 1 shows further details.

**Table 1:** Identification of articles.

ID	Author and year	Study design	Population	Control	Intervention	Intervention details
1	de l'Escalopier et al., 2019^11^	Retrospective cohort	377 (104 SCI)		Surgical resection	Surgical resection in symptomatic patients. Minimal resection to achieve functional ROM. Post-procedure physical therapy and pre-procedure ATB
2	Ploumis et al., 2015^12^	Retrospective control case	125	174	Alendronate	Administration of alendronate 70 mg per week, averaging 38.17 ± 57.89 weeks. The patients were followed for an average of 626.72 ± 620.49 days.
3	Ester et al., 2022^13^	Retrospective cohort	3 SCI and 1 CCT		Radiotherapy (8 Gy) + resection + NSAIDs Radiotherapy (8 Gy) + etidronate	Irradiated 3 hips, post resection, with 8 Gy, followed by 6 weeks of 25 mg of indomethacin, 3 times/day. Three hips and 2 elbows were irradiated + etidronate, no surgery.
4	Honore et al., 2020^14^	Retrospective control case	11 SCI and 8 CCT	76 (SCI or CCT)	Radiotherapy (7.5 Gy)	RT was performed with 7.5 Gy perioperatively to prevent recurrence.
5	Zakrasek et al., 2019^15^	Retrospective control case	27	81	NSAIDs	Use of NSAIDs (indomethacin 25 mg 3 times/day or celecoxib 200 mg/day) for 15 days or more. Followed up for an average of 63 days, being evaluated an average of 21 days after trauma.
6	Romero-Muñoz et al., 2018^16^	Retrospective cohort	20		Surgical resection + NSAIDs	Surgical resection of HO in 16 cases and Girdlestone in 4 cases. Goal of surgery: gain 90° of flexion and 20° of abduction. Physical therapy + celecoxib 200 mg/day for 4 weeks after the procedure.
7	Müseler et al., 2017^17^	Retrospective cohort	244		Radiotherapy (7 Gy)	Single radiation dose of 7 Gy in patients detected with HO (via biweekly US screening). After an average of 4.9 days after diagnosis, treatment was performed, which was on average 63.2 days after SCI.
8	Citak et al., 2016^18^	Retrospective cohort	13		Single radiation dose (7 Gy, 6 Gy in two cases)	Single radiation dose in patients with HO (via biweekly US screening).

HO: heterotopic ossification; SCI: spinal cord injury; ROM: range of motion ATB: antibiotic; CCT; craniocerebral trauma; NSAIDs; Non-steroidal anti-inflammatory drugs; US: ultrasound.

### Evaluation of interventions

We found that four studies dealt with surgical resection but only two evaluated it as a main treatment intervention, one of which with the association of NSAIDs for four weeks. ^(^ ^11^ ^),(^ ^16^ The others mainly evaluated the action of radiotherapy (RT) as an adjuvant. ^(^ ^13^ ^),(^ ^14^ Cases of surgical resection, mainly associated with the use of NSAIDs, showed low or even null recurrence rates, but had high rates of postoperative complications, infections being their main cause. Specifically, Honore et al. ^(^ ^14^ observed no statistical difference in HO recurrence between the group with resection associated with RT and that with resection only, but found significant infection rates in the group that performed RT (case group). Therefore, the study was unable to recommend this association, especially in cases of complicated recurrent HO, as were the majority (78.9%) of the case group - unlike the control group (5.3%).

**Figure 2:**
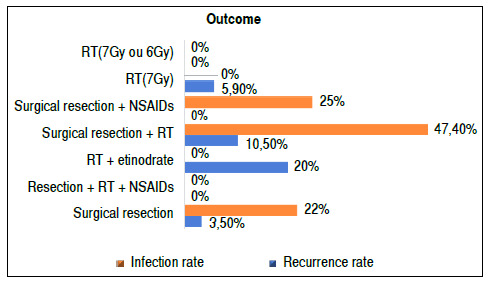
Outcome by intervention. RT: radiotherapy; NSAIDs: non-steroidal anti-inflammatory drugs.

Of the analyzed studies, three addressed RT as the main treatment, two in isolation^15^ ^),(^ ^16^ and one associated with etidronate. ^(^ ^13^ Recurrence rates were also low but generally higher than surgical resection (Figure 2). The study with the highest recurrence rate was the one that associated resection with etidronate. A patient showed disease progression, a high percentage due to their small sample. The advantage of RT is the near absence of complications, but all studies had short follow-ups. Furthermore, two studies on isolated RT performed early treatments due to ultrasound screening diagnoses (an institutional protocol) rather than by patients’ clinics.

Of the total, two studies addressed HO prevention methods, one on the use of alendronate and another on NSAIDs (celecoxib or indomethacin). They found no statistical difference in the use of alendronate to prevent HO. However, a reduction in alkaline phosphatase (ALP) was observed, which was high in the group that developed HO. ^(^ ^12^

In the study with NSAIDs, occurrence decreased by 28.4% after at least 15 days of treatment during the acute period after trauma. ^(^ ^15^ Table 2 shows further details on the outcomes of the studies.

**Table 2:** Outcomes of the interventions.

ID	Outcome	Complications	Limitations
1	Recurrence: 3.5%.	Intraoperative hemorrhage; femoral neck fracture, local infection (10.3% overall, 22% in those with spinal cord injuries)	
2	No statistical difference was observed for the prevention of HO. However, patients who developed HO had altered serum ALP levels, and the group taking alendronate was positively correlated with normal ALP levels.	No reported complications	Inhomogeneous group; no exact protocol for the treatment beginning and duration
3	Efficacy rate of 87.5% in total; 1 patient progressed to HO in the etidronate group.	Grade 2 dermatitis in a patient	The effect of radiation alone has not been proven. Small sample, short follow-up (11 weeks), unknown effect and long-term toxicity.
4	Case group: 10.5% of recurrence, 47% with postoperative complications. Control group: 5.3% of recurrence, 23.3% with postoperative complications. There was no statistical difference in the recurrence of HO between groups but a higher rate of postoperative complications in the study group.	Main complication: postoperative sepsis, especially in the case group.	Patients undergoing RT tended to recurrence (inhomogeneous groups). A total of 78.9% of case patients had HO recurrence, while only 5.3% in the control group.
5	A total of 28.4% reduction in the rate of occurrence of HO in patients who used NSAIDs for 15 days or more in the acute phase of rehabilitation after SCI. (indomethacin 75 mg/day or celecoxib 200 mg/day) p < 0.006.	No observed adverse effects or complications	Non-randomized choice and non-blinded patients. Average follow-up of only 60 days. Non-standardization of days of use of NSAIDs and date of introduction.
6	Recurrence: 0% Important ROM gain. Flexion 90°, abduction 20°, IR 20°, and ER of 40° on average. All patients were satisfied with the postoperative outcome, even those with complications. Minimum follow-up of 1 year.	Complication in 30%: Deep infections (3 cases); retained bruises (2 cases); intraoperative deep femoral lesion (1 case)	Small sample
7	Recurrence: 13 patients (5.3%), of which, 26 hips (5.9%). A further dose was repeated in these patients, in which only 1 patient developed ankylosis. On average, patients were followed for 89.4 days.	No observed complications	The lower recurrence rate may stem from two reasons: short follow-up and early treatment due to screening. Recurrence cases were based on clinical pictures, which allows for underdiagnosis.
8	Average follow-up: 88.8 days. ROM gain: 92.1° flexion, 94.5° abduction, 26.4° external rotation. Recurrence: 0%	No observed complications	Only screened people with symptoms, there may be underdiagnosis of HO recurrence. Short follow-up time.

HO: heterotopic ossification; ALP: alkaline phosphatase; NSAIDs; non-steroidal anti-inflammatory drugs; SCI: spinal cord injury; ROM: range of motion; RT: radiotherapy; ER: external rotation; IR: internal rotation.

## DISCUSSION

This systematic review aimed to investigate methods to prevent and treat neurogenic HO in people with spinal cord injuries.

The analyzed studies (all retrospective) ranged from level 3 to 4 of evidence. The main problem in most studies referred to their short post-treatment follow-up. HO may have recurred after these periods.

Resection surgery effectively treated HO, despite high infection rates. Romero-Muñoz’s^16^ patients felt satisfied with their outcome (including those who had complications, such as operative wound infections). Infection occurs particularly often in patients with SCI, as it disrupts neuroimmune regulation, causing a deficiency of peripheral immunity. Furthermore, constant vasodilation due to sympathetic denervation causes chronic inflammation in these patients, delaying their healing, especially in cases with pressure ulcers. ^(^ ^19^ ^),(^ ^20^

Radiotherapy has also shown promise, but it should be followed up for longer. The reviewed studies had short follow-ups. HO could have reoccurred in the medium term, as could have adverse effects in the long term. As mentioned, HO diagnosed by ultrasound screening received early treatment. This was an institutional protocol and patients may have had no complaint or clinic. Thus, we were unable to extrapolate this positive effect to more mature HO, chronic HO, or HO that developed ankylosis. The peak incidence of HO occurs in the first two to three months after SCI, ^(^ ^21^ configuring the window of opportunity to institute screening protocols and thus enable treatment with RT, which has a lower complication rate than surgery.

Radiotherapy associated with surgery in complicated cases of HO, such as those with recurrence or higher risk factors associated with recurrence, has shown to ineffectively prevent HO and increase the risk of local infections. Patients with spasticity, complete spinal cord injury (AIS A), urinary tract infections, pneumonia, and pressure ulcers were associated with a higher risk of developing HO. ^(^ ^22^ ^),(^ ^23^ In the presence of these factors, it is of paramount importance to treat possible comorbidities. These patients are also the greatest candidates for screening, enabling early treatment.

Regarding the studies on HO prevention, the use of NSAIDs proved to be effective, despite short follow-ups. ^(^ ^15^ Banovac et al. ^(^ ^24^ showed in 2001 the positive effects of using indomethacin to prevent HO in the first two months following the SCI. Later, in 2004, the use of rofecoxib (a selective COX-2 inhibitor) ^(^ ^25^ corroborated that positive result. The use of alendronate failed to prevent HO but it reduced ALP levels. Its heterogeneous study group and divergent protocols may have generated a bias. Thus, prospective studies with well-established protocols on the use of oral bisphosphonates could show positive results, considering the association found with ALP levels. However, the meta-analysis in Yolcu et al. ^(^ ^26^ in 2020 showed no preventive effect with the use of bisphosphonates, although NSAIDs obtained positive effects.

Our research found several case reports, which we excluded due to our exclusion criteria. However, they showed the benefits of extracorporeal shock therapy to reduce pain and improve range of motion, with promising results. ^(^ ^27^ ^),(^ ^28^

The inclusion of heterogeneous studies with small samples and retrospective studies are limitations of this study.

As strengths, we highlight the opportunity to gather the various methods of treatment and prevention in a single, carefully researched work to facilitate and guide the treatment of these cases.

## CONCLUSION

By analyzing the data in this systematic review, the use of NSAIDs proved to be the best method of prevention, if administered in the acute period after spinal cord injury.

For the already established HO treatment, the best method was surgical resection, being more effective if associated with the use of NSAIDs for four weeks after resection. But because of the high rate of postoperative complications, it is recommended to restrict this method to patients with a significant functional limitation, such as the inability to transfer from bed to wheelchair. Radiotherapy is promising in treating cases of HO detected early but not in association with surgical resection or in cases of recurrence.

Therefore, a good follow-up for these patients may include treating associated risk factors, such as urinary tract infection, tracheostomy care, and pressure injury, instituting NSAIDs protocols for the acute phase of SCI, and screening these patients at risk in the acute phase, as well as radiotherapy for early cases and resection for cases of ankylosis.
